#  Efficient, sparse biological network determination

**DOI:** 10.1186/1752-0509-3-25

**Published:** 2009-02-23

**Authors:** Elias August, Antonis Papachristodoulou

**Affiliations:** 1 Department of Engineering Science, University of Oxford, Parks Road, Oxford OX1 3PJ, UK

## Abstract

**Background:**

Determining the interaction topology of biological systems is a topic that currently attracts significant research interest. Typical models for such systems take the form of differential equations that involve polynomial and rational functions. Such nonlinear models make the problem of determining the connectivity of biochemical networks from time-series experimental data much harder. The use of linear dynamics and linearization techniques that have been proposed in the past can circumvent this, but the general problem of developing efficient algorithms for models that provide more accurate system descriptions remains open.

**Results:**

We present a network determination algorithm that can treat model descriptions with polynomial and rational functions and which does not make use of linearization. For this purpose, we make use of the observation that biochemical networks are in general 'sparse' and minimize the 1-norm of the decision variables (sum of weighted network connections) while constraints keep the error between data and the network dynamics small. The emphasis of our methodology is on determining the interconnection topology rather than the specific reaction constants and it takes into account the necessary properties that a chemical reaction network should have – something that techniques based on linearization can not. The problem can be formulated as a Linear Program, a convex optimization problem, for which efficient algorithms are available that can treat large data sets efficiently and uncertainties in data or model parameters.

**Conclusion:**

The presented methodology is able to predict with accuracy and efficiency the connectivity structure of a chemical reaction network with mass action kinetics and of a gene regulatory network from simulation data even if the dynamics of these systems are non-polynomial (rational) and uncertainties in the data are taken into account. It also produces a network structure that can explain the real experimental data of *L. lactis *and is similar to the one found in the literature. Numerical methods based on Linear Programming can therefore help determine efficiently the network structure of biological systems from large data sets. The overall objective of this work is to provide methods to increase our understanding of complex biochemical systems, particularly through their interconnection and their non-equilibrium behavior.

## Background

Determining the interaction topology in large-scale biological systems has been an active area of research for some time now. Most methodologies that deal with high-throughput experimental data make use of information about the behavior of the system locally around the steady-state [1-3]. For example, a class of techniques that fall under the rubric of 'stationary state Jacobian Matrix Elements' analyzes the system behavior when it is perturbed locally from steady-state and looks at whether the concentration of one species is increased or decreased when another species' concentration is increased. In [4] and [5], the authors have built on this approach and determined the functional interactions in cellular signaling and gene networks by taking into account the 'modular' structure of the networks in question. Alternatively, inferences about the topology of the network can be made by introducing pulse changes in concentration of a chemical species in the network, and observing the network's response, concluding causal chemical connectivities [6]. In [3], a linear dynamical system was considered to represent a gene regulatory network, and an approach, based on Linear Programming, was proposed in order to obtain the sparsest network structure from genetic perturbation experiments.

However, most information of the system dynamics is in its transient behavior and, more importantly, many reactions that the living cell requires are actually for most of the time far from steady state [7]. The problem of determining the network structure in the case where transient time-series data for non-equilibrium behavior are available is much harder and this has been an active area of research for over a decade. The reason is that while such data are often abundant due to the development of high-throughput, effective experimental techniques, at the same time, a high computational effort is required to extract information about the network structure using traditional techniques. A recent review of available techniques can be found in [8] or [9], but earlier articles, such as [10], also list several approaches to this network identification problem.

In actual fact, identifying the interconnection topology in biological and biochemical systems is of greater importance than extracting the parameters (the rates of the various reactions) that best fit the particular time series data. There are several reasons for this: first, the parameters are often collected under noisy experimental conditions and are sensitive to the laboratory environment. Second, as is often the case with large networks, persistence of observed phenomena is robust to a large range of most parameter values and therefore identifying these parameters exactly is not of great interest. Indeed, *chemical reaction networks *often have the same functionality in the neighborhood of most of the nominal reaction rates. But most importantly, networks are rarely robust to the random rewiring of the underlying interconnection structure and hence determining the network connectivity is much more important than determining the kinetic parameters that fit the particular data.

In this paper, we first consider chemical reaction networks with *mass action kinetics *(see references [11] and [12]) and seek to identify the chemical pathways and mechanisms, that is, how the chemical complexes interact within the chemical network. Chemical reaction networks are dynamical systems of the form

(1) $$\dot{x}=Af(x),\;x\in {\mathbb{R}}^{n},\;A\in {\mathbb{R}}^{n\times m},$$

where the unknown matrix encompassing the connectivity structure is *A *and the vector of functions *f*: ℝ^*n *^→ ℝ^*m *^(which need to satisfy appropriate smoothness conditions to ensure local existence and uniqueness of solutions) is known. This makes (1) linear in the unknown parameters. Our main objective is to provide a procedure to identify the interconnection topology that is encapsulated in *A*, given experimental time-series data.

An important property of the network given by *A *is sparseness, i.e., it has much less edges than the full graph with the same vertex set. In this paper, we extend the results in [13] that focus on obtaining sparse interconnection networks from experimental data to general and large-scale networks. We apply the presented methodology in order to reconstruct a biochemical network from mock-up experimental data obtained through simulations. More importantly, we show its validity in determining the glycolytic pathway of *Lactococcus lactis *from real experimental data. Although this pathway has been investigated in great detail (see for example, [14-16]) and is the test object of many system identification approaches as a recent paper fittingly notes in its title, it is 'an unfinished systems biological case study' [14].

Finally, we suggest how the method of identifying connectivity for systems of the form (1) can be adjusted to determine the structure of *gene regulatory networks*, in which the unknown parameters do not enter the system dynamics in an affine way. We then apply the methodology in order to reconstruct a gene regulatory network from mock-up experimental data obtained through simulations.

## Notation

ℝ, ℝ^*n*^, ℝ^*m *× *n *^is the set of all real numbers, real vectors of length *n*, *m *× *n *real matrices

*A*_*ij *_(*i*, *j*)th is the (*i*, *j*) entry of matrix *A *∈ ℝ^*m *× *n*^

${\mathbb{R}}_{+}^{n},{\bar{\mathbb{R}}}_{+}^{n}$ :{*x *∈ ℝ^*n*^: *x*_*i *_> 0, *i *= 1, ..., *n*}, {*x *∈ ℝ^*n*^: *x*_*i *_≥ 0, *i *= 1, ..., *n*}

vec(*A*) is a vector which contains the columns of *A *stacked one below each other

*e *=[1, 1, ⋯, 1]^T^

diag(*A*), *A *∈ ℝ^*n *× *n *^is a vector of length *n*, where (diag(*A*))_*i *_= *A*_*ii*_

diag(*x*), *x *∈ ℝ^*n *^is a matrix in ℝ^*n *× *n*^, where (diag(*x*))_*ii *_= *x*_*i *_and (diag(*x*))_*ij *_= 0 if *j *≠ *i*

## Methods

### Chemical reaction networks

Chemical reaction networks are used to describe and understand biological processes. An illustrative example is the following reaction network proposed by Michaelis and Menten for chemical reactions involving enzymes,

(2) $$E+S\overset{k_{1}}{\underset{k_{-1}}{⇄}}ES\overset{k_{2}}{\underset{k_{-2}}{⇄}}E+P.$$

Here, *S *denotes the substrate, *E *the enzyme, *ES *the enzyme-substrate complex and *P *the final product. They are the so called *species *that participate in the reactions. The positive constants *k*_1_, *k*_-1_, *k*_2 _and *k*_-2 _are the reaction *rate constants*, *edges *represent reactions and *vertices *represent *complexes *(the objects that appear before and after the reaction arrows).

In *chemical kinetics*, it is common to assume that the dynamics of the chemical reaction network (such as the one given by (2)) can be described by the following set of nonlinear ODEs [17]:

(3) $$\frac{dx}{dt}≜\dot{x}=Nv(x),$$

where *v*(*x*) is the *rate vector *(or *flux vector*), *x *is the concentration vector of the different species and *N *is the *stoichiometric matrix*. If *p *molecules of species *i *appear before the reaction arrow in reaction *j *then *N*_*ij *_= -*p *and if they appear after then *N*_*ij *_= *p*.

The description given by (3) hides the underlying chemical network structure, which we aim to determine in this paper. Hence, in the following, we introduce the notation used in *chemical reaction network theory*, which decomposes *N *and *v*(*x*) into: the so called *bookkeeping matrix Y*, which maps the space of complexes into the space of species; the concentration vector of the different complexes Ψ(*x*); and matrix *A*_*κ*_, which defines the network structure. For the Michaelis-Menten reaction, the vectors of species and complexes are given by

$$x=\left[\begin{array}{c} \left[E\right]\\ \left[S\right]\\ \left[ES\right]\\ \left[P\right] \end{array}\right]\text{ and }\Psi (x)=\left[\begin{array}{c} \left[E][S\right]\\ \left[ES\right]\\ \left[E][P\right] \end{array}\right],$$

respectively. The elements of the *i*th row of matrix *Y *tell us in which complexes species *i *appears and how often; or, equivalently, the entries to the *j*th column tell us of how much of each species complex *j *is made of. For (2),

$$Y=\left[\begin{array}{ccc} 1 & 0 & 1\\ 1 & 0 & 0\\ 0 & 1 & 0\\ 0 & 0 & 1 \end{array}\right].$$

Matrix *K *is the transpose of the *weighted adjacency matrix *of the *digraph *representing the chemical reaction network; that is, entry *K*_*ij *_is nonnegative and corresponds to the rate constant associated with the reaction from complex *j *to *i*. The so called *kinetic matrix A*_*κ *_is given by *A*_*κ *_= *K *- diag(*K*^T^*e*). For (2),

$$K=\left[\begin{array}{ccc} 0 & k_{-1} & 0\\ k_{1} & 0 & k_{-2}\\ 0 & k_{2} & 0 \end{array}\right]\text{ and }A_{\kappa }=\left[\begin{array}{ccc} -k_{1} & k_{-1} & 0\\ k_{1} & -(k_{-1}+k_{2}) & k-2\\ 0 & k_{2} & -k_{-2} \end{array}\right].$$

In chemical reaction network theory, it is common to assume mass action kinetics. The law of mass action assumes that if reactions take place at constant temperature in a homogenous and well mixed solution then the probability of a collision between molecules is proportional to the product of their concentrations. This means that ln Ψ(*x*) = *Y*^T ^ln *x*, and one reformulates the set of nonlinear ODEs given by (3) as [18]:

(4) $$\dot{x}=YA_{\kappa }\Psi (x).$$

In general, we assume that a chemical reaction network has *n *species and *m *complexes. Thus, in (4): *x *∈ ${\bar{\mathbb{R}}}_{+}^{n}$, Ψ(*x*) ∈ ${\bar{\mathbb{R}}}_{+}^{m}$, *A*_*κ *_∈ ℝ^*m *× *m*^, and $Y\in {\bar{\mathbb{R}}}_{+}^{n\times m}$. Experimental data is stacked in vector Ψ(*x*) and often matrix *Y *is known such that we can explicitly search for the network structure given by *A*_*κ*_. Finally, for clarity, we provide the expanded ODE representation of the Michaelis-Menten reaction given by (2):

(5) $$\begin{array}{c} {}[\dot{E}]=-k_{1}[E][S]+(k_{-1}+k_{2})[ES]-k_{-2}[E][P],\\ {}[\dot{S}]=-k_{1}[E][S]+k_{-1}[ES],\\ {}[\dot{E}S]=k_{1}[E][S]-(k_{-1}+k_{2})[ES]+k_{-2}[E][P],\\ {}[\dot{P}]=k_{2}[ES]-k_{-2}[E][P]. \end{array}$$

### Determining affine and sparse interconnections in dynamical systems

Consider a dynamical system of the following form:

(6) $$\dot{x}=Af(x),\;x\in {\mathbb{R}}^{n},\;A\in {\mathbb{R}}^{n\times m},$$

where *f*(·) ∈ ℝ^*m *^is a vector of known functions, which satisfy appropriate smoothness conditions to ensure local existence and uniqueness of solutions. With *A *= *Y A*_*κ *_and *f *(*x*) = Ψ(*x*), the above description results in a dynamical system of the form given by (6). Note that the unknown parameters (which also encode the network structure) are in *A*, which enters the system dynamics linearly. Let neither the value of the entries nor the structure of matrix *A *be known. What we wish to find is the structure and entries in matrix *A*, given experimental data.

For this purpose, we consider the following discrete-time system:

(7) *x*(*t*_*k *+ 1_) = *x*(*t*_*k*_) + (*t*_*k *+ 1 _- *t*_*k*_) *Af*(*x*(*t*_*k*_)),

which is the Euler discretization of (6).

Now, the set of measurements, which we denote by $\hat{x}$, can be used to fit the unknown entries to *A *such as to minimize the error between the data and the model predictions, which are given by (7). It is popular to solve the minimization problem which results in the least 2-norm of the error between *x*_*i*_(*t*_*k*+1_) and ${\hat{x}}_{i}(t_{k+1})$ (least squares minimization problem). We can write such an error metric as:

(8) min||*Ma *- *b*||_2_

where *a *∈ ℝ^*nm *^is a vector containing *A*_*ij*_, which we treat as decision variables, and *M *∈ ℝ^((*p*-1) × *n*) × *nm *^and *b *∈ ℝ^(*p*-1) × *n *^are defined by 'stacking' all such conditions obtained by manipulating the data as per (7). Here *p *corresponds to the number of measurements. This problem has the following analytical solution:

(9) a* = *M*^†^*b *≜ (*M*^T ^*M*)^-1^*M*^T^*b*

There are a few drawbacks of the above least-squares approach. In the presence of extra constraints on the variables *A*_*ij *_(constrained regression), the problem does not have a closed-form solution, in general. Such constrained minimizations, the simplest of which is a *Second Order Cone Problem *(SOCP) [19], may carry a significant computational cost for large problems. The same is true if the data are contaminated with error which needs to be taken into account when producing *A*_least-squares _[20]. Furthermore, the solution to a least-squares problem will not be sparse in general; it will rather result in a full matrix.

In [19] and more recently in [21], the fact was mentioned that a large number of elements of the solution *z *of a *Linear Program *(LP) such as

(10) min ||*z*||_1_,

are zero, that is, (10) produces sparse solutions. For this reason, this is the approach we follow in the paper. In particular, if *A *is sparse then the following program seeks explicitly to minimize the entries to matrix *A *and, thus, tries to recover this property of the matrix:

(11) $$\begin{array}{ll} \min & ||\text{vec}(A)|{|}_{1}\\ \text{s}\text{. t}\text{.} & -\mu_{k}^{-}\leq -\hat{x}(t_{k+1})+\hat{x}(t_{k})+(t_{k+1}-t_{k})Af(\hat{x}(t_{k}))\leq \mu_{k}^{+},\\ & \mu_{k}^{+}\geq 0,\;\mu_{k}^{-}\geq 0,\;\forall k,\;k=1,...,p-1. \end{array}$$

By making $\mu_{k}^{+}$ and $\mu_{k}^{-}$ as small as possible for all *k*, we can ensure that the data are in close Euler-fit with the model making the approximation error as small as possible. The advantage of solving LPs is that the task can be performed using fast, efficient and readily available algorithms. Note also that the number of decision variables in (11) relates directly to the size of *A *and not of the data, which makes it suitable for the identification of large-scale systems. Support for the validity of above heuristic to obtain a sparse interconnection matrix *A *are also Theorem 1.1 of [22] and the work presented in [23].

An additional advantage of our approach is also that we may incorporate uncertainties in the measurements with little additional computational complexity. If we model the uncertainty in the measurements as

(12) $$\tilde{x}(t_{k})-\epsilon (k)\leq \hat{x}(t_{k})\leq \tilde{x}(t_{k})+\epsilon (k),\;\tilde{f}(t_{k})-\delta (k)\leq f(\hat{x}(t_{k}))\leq \tilde{f}(t_{k})+\delta (k),\;\epsilon (k),\delta (k)\geq 0,$$

$\tilde{x}$ (*t*_*k*_) ≥ 0, $\tilde{f}$ (*t*_*k*_) ≥ 0, for all *k*, and *A*_*ij *_≥ 0 then we can formulate the robust counterpart to (11) that is still an LP (see also [24,25]). The following LP is a robust formulation of program (11):

(13) $$\begin{array}{ll} \min & ||\text{vec}(A)|{|}_{1}\\ \text{s}\text{. t}\text{.} & -\mu_{k}^{-}\leq -\tilde{x}(t_{k+1})-\epsilon (k+1)+\tilde{x}(t_{k})-\epsilon (k)+(t_{k+1}-t_{k})A\left(\tilde{f}(t_{k})-\delta (k)\right),\\ & -\tilde{x}(t_{k+1})+\epsilon (k+1)+\tilde{x}(t_{k})+\epsilon (k)+(t_{k+1}-t_{k})A\left(\tilde{f}(t_{k})+\delta (k)\right)\leq \mu_{k}^{+},\\ & A_{ij}\geq 0,\;\forall i,j,\;\tilde{x}(t_{k}),\tilde{f}(t_{k}),\epsilon (k),\delta (k),\mu_{k}^{+},\mu_{k}^{-}\geq 0,\;\forall k,\;k=1,...,p-1. \end{array}$$

In summary, using the above ideas, we aim to extract from data the sparsity pattern in matrix *A*, which is related to the connectivity of the underlying graph structure, drawing conclusions on the network interaction topology.

Finally, note that if data points are rare, that is *p *≤ *m*, and there are not any constraints on matrix *A *then the error between the data and the model predictions can be made zero and (9) does not have a unique solution. However, the following LP can be used to try to recover the sparsity structure of the matrix:

(14) $$\begin{array}{ll} \min & ||\text{vec}(A)|{|}_{1}\\ \text{s}\text{. t}\text{.} & \hat{x}(t_{k+1})=\hat{x}(t_{k})+(t_{k+1}-t_{k})Af(\hat{x}(t_{k})),\;\forall k,\;k=1,...,p-1. \end{array}$$

### Obtaining the structure of a gene regulatory network

Using the same ideas, another class of a networks that can be determined are gene regulatory networks given microarray time-series data. We first briefly explain the function of gene regulatory networks and DNA microarray time-series.

**Figure 1:**
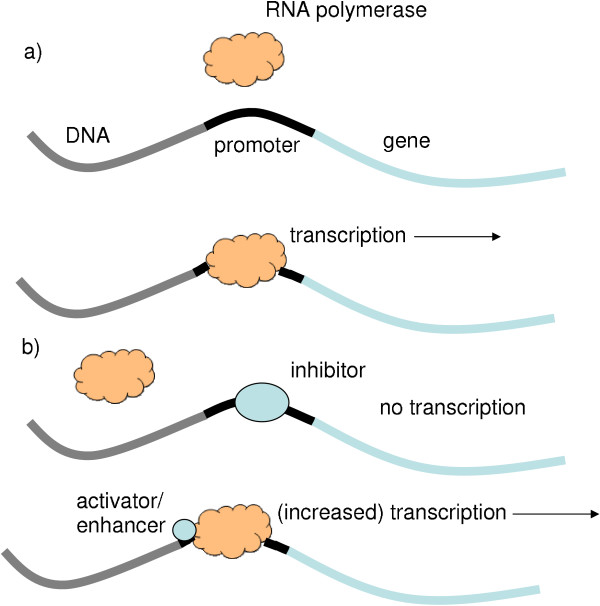
**Diagram showing the process of transcription**. a) The RNA polymerase binds to the promoter sequence on the DNA and transcribes a gene. b) Transcription can be controlled by inhibitors or activators acting at the promoter sequence.

A gene encodes the information necessary to produce a specific protein. The process, in which the information is used to synthesize a protein, is highly controlled and this control allows the cell to vary the level of a particular protein in the cell depending on the cell's need for this protein. The first step of synthesizing a protein from a gene is RNA polymerase transcribing gene information from DNA to mRNA (see Figure 1a). This mRNA is then translated into synthesized proteins by ribosomes. Control can occur at a number of stages including the synthesis of mRNA, subsequent processing of the mRNA, control of the ribosome and control of mRNA stability. Some proteins, called transcription factors, are responsible for the regulation of the initiation of transcription. A transcription factor binds to the DNA, at the promoter site, in order to either inhibit or activate (or alternatively increase the rate of) the transcription of mRNA that is responsible for the synthesis of a specific protein (see Figure 1b). (Note that self regulation is also possible.) The collection of DNA segments which interact with each other in the manner described is called the gene regulatory network.

In order to understand the dynamics and behavior of a gene regulatory network, three levels of information are required:

1. The network interconnection in the form of a *directed graph*;

2. Whether an edge from node *i *to node *j *means that transcription factor *i *is activating (denoted by arrow) or repressing (denoted by 'hammer') *j*;

3. The activation/repression rates for the transcription factors.

Time-series obtained from DNA microarrays [26,27] are extremely helpful to obtain the structure of gene regulatory networks. This is because DNA microarrays allow observation of the presence of specific mRNA within the cell and in particular, time-series data allow measurements on how these change over time after a perturbation, or when following the cell cycle.

Now, consider the model of a gene regulatory network as described in [28] and [29], where nodes represent genes. Knowledge of the three hierarchal levels of information mentioned previously is necessary to describe the network. The first level determines whether there is an interaction between proteins *X*_1 _and *X*_2_. An interaction of the form '*X*_1 _→ *X*_2_' means that protein *X*_1 _activates the production of protein *X*_2 _and '*X*_1 _⊣ *X*_2_' that *X*_1 _inhibits it. This information belongs to the second level. The activation and repression Hill input functions are given mathematically by (see [28]):

(15) $$\frac{kx_{1}^{n}}{1+kx_{1}^{n}},\text{ and }\frac{1}{1+kx_{1}^{n}},$$

respectively, where *x*_1 _is the concentration of *X*_1_. (In [29], the notation $\frac{1}{K}$ is used instead of *k*. For clarity, we have adopted a 'simpler' notation.) Knowledge about the magnitude of activation or repression coefficient *k*, *k *> 0, and exponent *n*, *n *> 0, is part of the third level of information.

If there exists more than one connection to a particular gene/node then all transcription factors associated with the connections could be necessary to fulfill a specific task (activation or inhibition) or it might be that any of them is sufficient to have an effect on the transcription process; more complex combinations are also possible. In [28,30], a generalized input function of the following form is presented, which takes the possible interplay of different transcription factors into account:

(16) $$f_{i}(x)=\frac{\sum_{j}b_{ij}x_{j}^{n_{ij}}}{1+\sum_{j}k_{ij}x_{j}^{m_{ij}}}.$$

Here, activation of protein *X*_*i *_by protein *X*_*j *_is represented by *n*_*ij *_= *m*_*ij *_> 0, and repression by *n*_*ij *_= 0, *m*_*ij *_> 0. The contribution of the different transcription factors on the transcription rate is denoted by *b*_*ij*_. Putting everything together, the mathematical description of the dynamics of the concentrations of protein *X*_*i *_of an arbitrarily large gene regulatory network is as follows:

(17) $${\dot{x}}_{i}=\gamma_{i}+f_{i}(x)-d_{i}x_{i},$$

where *γ*_*i *_> 0 is the basal transcription production rate and *d*_*i *_> 0 is the degradation/dilution rate. In the above model, however, the vector field (right hand side of Equation (17)) is not affine in the unknown parameters and therefore one may think that the method proposed in the previous section can not be extended for this case; we show here how the above can be reformulated and cast in a form that allows identification using Linear Programming.

Let Δ*t *= *t*_ℓ+1 _- *t*_ℓ_. A discrete-time system that approximates (17) is:

(18) *x*_*i*_(*t*_ℓ+1_) = *x*_*i*_(*t*_ℓ_) + Δ*t*(*γ*_*i *_+ *f*_*i*_(*x*_*i*_(*t*_ℓ_)) - *d*_*i*_*x*_*i*_(*t*_ℓ_)).

Indeed, if *b*_*ij*_, *k*_*ij *_and *m*_*ij *_are unknown then (18) is not affine in the unknown parameters as is the case in (7). We rewrite (18) as follows:

(19) $$(x_{i}(t_{\ell })(1-\Delta td_{i})-x_{i}(t_{\ell +1})+\Delta t\gamma_{i})(1+\sum_{j}k_{ij}x_{j}^{m_{ij}})+\Delta t\sum_{j}b_{ij}x_{j}^{{\tilde{n}}_{ij}}+\Delta tb_{i}=0.$$

In (19), if *n*_*ij *_= 0 then we denote it by ${\bar{n}}_{ij}$ and let $b_{i}=\sum_{j}b_{ij}x_{j}^{{\bar{n}}_{ij}}=\sum_{j}b_{ij}$. If *n*_*ij *_> 0 then we denote it by ${\tilde{n}}_{ij}$. For all *i*, *j*, let an entry to matrix *B *be *b*_*ij *_for which *n*_*ij *_> 0, and let an entry of matrix *K *be *k*_*ij*_. As before, given a set of measurements, which we denote by $\hat{x}$, this set can be used to approximate the structure of the gene regulatory network determined by *b*_*ij*_, *b*_*i *_and *k*_*ij *_if the Hill coefficients *m*_*ij *_(and, thus, *n*_*ij*_) are known and the basal production and degradation rates are known or considered uncertain but within a known range. For instance, we can try to recover *B, K *through a LP. The following LP puts emphasis on minimizing the 1-norm of vec(*B*), *b*, and vec(*K*), which are independent of each other, while we keep the Euler discretisation error, *μ*, as small as possible.

(20) $$\begin{array}{ll} \min & ||\text{vec}([B\;b\;K])|{|}_{1}\\ \text{s}\text{. t}\text{.} & -\mu <({\hat{x}}_{i}(t_{\ell })(1-\Delta td_{i})-{\hat{x}}_{i}(t_{\ell +1})+\Delta t\gamma_{i})(1+\sum_{j}k_{ij}{\hat{x}}_{j}^{n_{ij}}+\Delta t\sum_{j}b_{ij}{\hat{x}}_{j}^{n_{ij}}+\Delta tb_{i}<\mu ,\\ & \mu >0,\;b_{ij}\geq 0,\;k_{ij}\geq 0,\;b_{i}\geq 0,\;\forall i,j,\ell (0\leq \epsilon_{1i}\leq \gamma_{i}\leq \epsilon_{2i},0\leq \varepsilon_{1i}\leq d_{i}\leq \varepsilon_{2i},\forall i). \end{array}$$

(The latter requirements in brackets correspond to the case of uncertain production and degradation rates.) Note that as per (16)

(21) *k*_*ij *_= 0 if and only if *b*_*ij *_= 0 or *b*_*i *_= 0, ∀*i*, *j*.

The following remark deals with the case when the solution of (20) violates (21). The rationale behind the idea is that by following these rules we can determine unambiguously whether activation or repression takes place.

**Remark 1 ***Since requirement (21) cannot be implemented in a LP, we deduce the following from the solution of (20) about the connectivity of the network when (21) is violated:*

- *if k*_*ij *_≠ 0, *b*_*ij *_= 0 *and b*_*i *_= 0 *then the production of X*_*i *_*is not affected by X*_*j*_; *that is, it is the same case as when k*_*ij *_= 0,

- *if b*_*ij *_≠ 0 *and k*_*ij *_= 0 *then X*_*j *_*enhances the production of X*_*i*_; *i. e., it is the same case as when k*_*ij *_≠ 0,

- *if b*_*i *_≠ 0 *and k*_*ij *_= 0 *for all i then the production of X*_*i *_*is not affected by X*_*j*_; *that is, it is the same case as when b*_*i *_= 0.

## Results and discussion

### Numerical experiments

#### An artificial chemical reaction network

**Figure 2:**
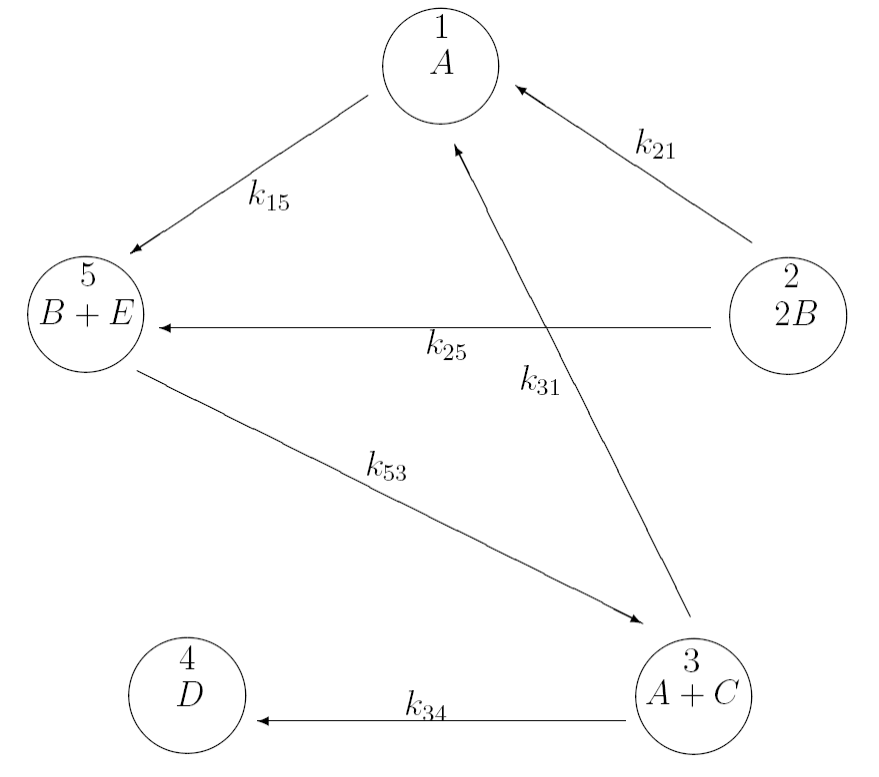
**A chemical reaction network**.

Consider the network with 5 species S = {*A*, *B*, *C*, *D*, *E*} and 5 complexes, C = {*A*, 2*B*, *A *+ *C*, *D*, *B *+ *E*} in Figure 2. Suppose we are given time series data for all the species in this system, but we do not know the topology of the interconnection. The first experiment examines the recovered interconnection diagram using the (non-robust) LP (11). Later on, we will consider the same problem with missing data on one species and a robust network determination problem.

We have implemented the network shown in Figure 2 with a *K *matrix of the form:

(22) $$K=\left[\begin{array}{ccccc} 0 & 0 & 0 & 0 & 0.8492\\ 0.3386 & 0 & 0 & 0 & 0.4290\\ 0.8244 & 0 & 0 & 0.0563 & 0\\ 0 & 0 & 0 & 0 & 0\\ 0 & 0 & 0.7364 & 0 & 0 \end{array}\right]$$

and have simulated the system with uniformly distributed initial conditions. The data sets were obtained by simulating the above set of nonlinear equations using SIMULINK. Ten such data sets were generated and incorporated in the LP.

Since we do not know how the chemical network is connected, and we cannot even speculate how parts of it may be connected, we need to assume a general structure for it and write the dynamics for the complete network. A least-squares approach, would yield the following structure in matrix *K*, where non-zero entries denote the fractional occurrences of non-zero *k*_*ij*_'s for the 10 data sets:

$$\left[\begin{array}{ccccc} 0 & 0.1 & 1 & 0.1 & 1\\ 1 & 0 & 0.8 & 0.2 & 1\\ 1 & 0.6 & 0 & 1 & 0.9\\ 0.1 & 0.8 & 0.9 & 0 & 0\\ 1 & 0.9 & 1 & 0.9 & 0 \end{array}\right]$$

Essentially the only zero element predicted is *k*_45_. Note that the diagonal of this matrix does not enter into our optimization. We write these entries as zero, but this is merely a convenient notational place holder. The resulting structure of the *K *matrix from our linear programming approach is given by

$$\left[\begin{array}{ccccc} 0 & 0 & 1 & 0 & 1\\ 1 & 0 & 0.5 & 0.4 & 0.9\\ 1 & 0 & 0 & 1 & 0.2\\ 0 & 0 & 0.8 & 0 & 0\\ 0.1 & 0 & 1 & 0.2 & 0 \end{array}\right]$$

where again non-zero entries denote the fractional occurrences of non-zero entries for the 10 data sets tested. Observe that the second column is equal to zero which implies that the second complex is not the product of any reaction. Having determined this sparse structure for the system, we can repeat the same LP optimization, but now impose the new information about the sparse structure obtained in the new linear program, i.e. that *k*_12 _= 0 etc. Iterating once on this data, we get the following results:

$$\left[\begin{array}{ccccc} 0 & 0 & 1 & 0 & 1\\ 1 & 0 & 0.5 & 0.3 & 0.7\\ 1 & 0 & 0 & 1 & 0\\ 0 & 0 & 0.8 & 0 & 0\\ 0 & 0 & 0.8 & 0 & 0 \end{array}\right]$$

This experiment reveals that the sparsity structure can be further reduced by an iterative procedure. One could also use the above as a 'probability' lookup table, and investigate sparsity structures, such as setting *k*_23 _and *k*_24 _equal to zero. Indeed this solution is also feasible, which reveals additional structure in the matrix *K*. Working this way, we have found that the following non-zero matrix results in feasible LPs:

$$K_{\text{nom}}=\left[\begin{array}{ccccc} 0 & 0 & k_{13} & 0 & k_{15}\\ k_{21} & 0 & 0 & 0 & k_{25}\\ k_{31} & 0 & 0 & k_{34} & 0\\ 0 & 0 & 0 & 0 & 0\\ 0 & 0 & k_{53} & 0 & 0 \end{array}\right]$$

which is the same as the network shown in Figure 2, but for a link between complex 1 and complex 3. Of course, this is not surprising: there is no unique reaction mechanism that can fit a data set; rather, there can be many networks which with some kinetic parameters can represent the same data within experimental error. In fact, we can only hope to *invalidate *a postulated reaction mechanism using data, a point we will return to in the concluding section.

The next experiment we performed was to assume that some of the species could not be observed in the experiments for technical reasons. In particular, we assumed that the concentration of species *A *could not be measured. This does not pose significant problems, as we can replace the occurrences of the terms in the vector field involving the variable *x*_1 _with a vector of new variables *q *which we also ask to be 'sparse', through minimization of the sum of *q*_*i*_. Eight such substitutions need to be made; the result is a matrix of the form:

$$K_{\text{miss}}=\left[\begin{array}{ccccc} 0 & 0 & 0 & 0 & 0\\ k_{21} & 0 & k_{23} & 0 & k_{25}\\ 0 & 0 & 0 & 0 & 0\\ 0 & 0 & k_{43} & 0 & 0\\ 0 & 0 & k_{53} & k_{54} & 0 \end{array}\right]$$

and a *q *= [*q*_1_, ..., *q*_8_] which corresponds to nonzero entries for *k*_31_, *k*_34_, *k*_35 _*k*_13 _and *k*_15_. Therefore in this case too, a sparse topology interconnection is obtained, but the matrix in this case is not as sparse as before.

Suppose now that data are uncertain, and we want to search for *robust *sparse structures for the *K *matrix. We set $\epsilon_{i}^{+}$ = $\epsilon_{i}^{-}$ = 0.0004 for *i *= 1, ..., 5 and all data points – such uncertainty could be due to roundoff errors (see Equation (12)). A robust LP can be formulated, as discussed earlier, and the obtained optimization results in a network with a richer sparsity structure:

$$K_{\text{rob}}=\left[\begin{array}{ccccc} 0 & 0 & k_{13} & 0 & k_{15}\\ k_{21} & 0 & k_{23} & 0 & k_{25}\\ k_{31} & 0 & 0 & k_{34} & 0\\ 0 & 0 & k_{43} & 0 & 0\\ 0 & 0 & k_{53} & 0 & 0 \end{array}\right]$$

Finally, we note that once a candidate network is determined, we can perform a least-squares minimization to obtain the best *k *values for a particular sparsity structure. For example, if we choose *K*_nom _as the sparsity structure and fit the least squares error over all 10 experiments, we get the following *K *matrix:

(23) $$K=\left[\begin{array}{ccccc} 0 & 0 & 0.0364 & 0 & 0.7721\\ 0.3295 & 0 & 0 & 0 & 0.3999\\ 0.7804 & 0 & 0 & 0.0553 & 0\\ 0 & 0 & 0 & 0 & 0\\ 0 & 0 & 0.6668 & 0 & 0 \end{array}\right]$$

**Figure 3:**
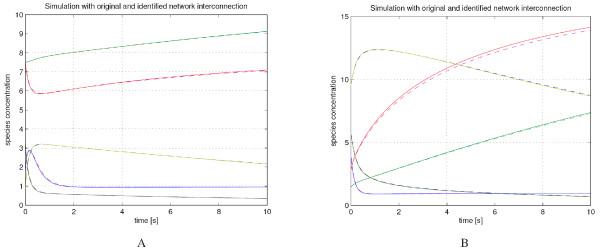
**Simulation of chemical reaction networks**. Simulation of the network with reaction rates (22) (solid line) and with reaction rates given by (23) (dashed line) from two initial conditions.

In figures 3A and 3B we show how the nominal system, with the *K *matrix given by Equation (22) compares in simulation with the *K *matrix given by Equation (23) for different initial conditions. We see that some initial conditions have better behavior for the two parameter sets than others. There is hope, however, that using other methods and through choice of a particular initial condition we can distinguish between the two network structures; the initial condition in Figure 3B shows some deviation in the dynamics of *x*_1_, and designing an experiment that would yield 'maximum' deviation would allow for differentiability between various models that can explain the same data. More details about this approach can be found in [31].

#### A sample gene regulatory network

Consider the artificial gene regulatory network modeled through

(24) $$\begin{array}{c} {\dot{x}}_{1}=\gamma_{1}-d_{1}x_{1},\\ {\dot{x}}_{2}=\gamma_{2}+\frac{b_{12}x_{1}}{1+k_{12}x_{1}}-d_{2}x_{2},\\ {\dot{x}}_{3}=\gamma_{3}+\frac{b_{43}x_{4}+b_{13}x_{1}+b_{3}}{1+k_{43}x_{4}+k_{13}x_{1}+k_{53}x_{5}}-d_{3}x_{3},\\ {\dot{x}}_{4}=\gamma_{4}+\frac{b_{54}x_{5}}{1+k_{54}x_{5}}-d_{4}x_{4},\\ {\dot{x}}_{5}=\gamma_{5}+\frac{b_{15}x_{1}+b_{5}}{1+k_{15}x_{1}+k_{25}x_{2}}-d_{5}x_{5}, \end{array}$$

where

$$B=\left[\begin{array}{ccccc} 0 & 0.51 & 0.87 & 0 & 0.80\\ 0 & 0 & 0 & 0 & 0\\ 0 & 0 & 0 & 0 & 0\\ 0 & 0 & 0.20 & 0 & 0\\ 0 & 0 & 0 & 0.22 & 0 \end{array}\right],K=\left[\begin{array}{ccccc} 0 & 0.31 & 0.87 & 0 & 0.15\\ 0 & 0 & 0 & 0 & 0.77\\ 0 & 0 & 0 & 0 & 0\\ 0 & 0 & 0.97 & 0 & 0\\ 0 & 0 & 0.79 & 0.44 & 0 \end{array}\right],$$

**Figure 4:**
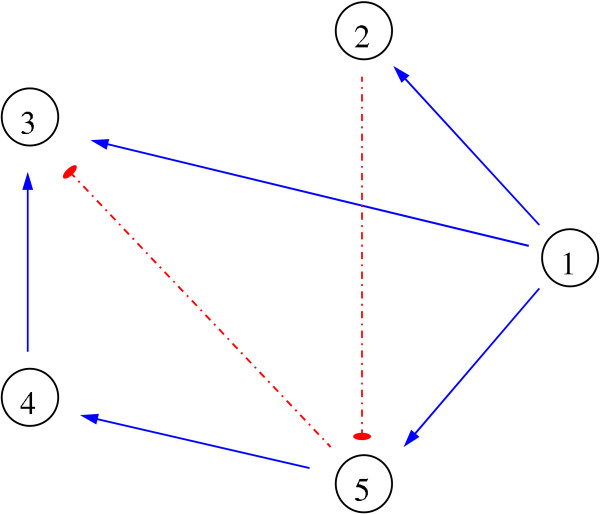
**The artificial gene regulatory network modeled through (24)**. Solid lines with an arrow head denote activation and dash pointed lines with a hammer head denote inhibition.

*b*_3 _= 0.71, *b*_5 _= 0.80, *γ*_*i *_= 0.1 and *d*_*i *_= 1. The network is depicted in Figure 4, where solid lines with an arrow head denote activation and dash pointed lines with a hammer head denote inhibition.

We assume that *d*_*i *_is known and *γ*_*i *_= *γ *for all *i*, where 0.095 ≤ *γ *≤ 0.105. We take 'measurements' every Δ*t *= 0.05 between *t *= 0 and *t *= 5 (time is in arbitrary units) from four different random initial conditions between 0 and 1 in order to obtain mock-up data. Solving (20) using the solver SeDuMi [32], we obtain the following results for matrices *B *and *K*:

$$B=\left[\begin{array}{ccccc} 0 & 0.48 & 0.22 & 0 & 1.15\\ 0 & 0 & 0 & 0 & 0\\ 0 & 0 & 0 & 0 & 0\\ 0 & 0 & 0 & 0 & 0\\ 0 & 0 & 0 & 0.11 & 0 \end{array}\right],K=\left[\begin{array}{ccccc} 0 & 0 & 0 & 0 & 0.61\\ 0 & 0 & 0 & 0 & 0.75\\ 0 & 0 & 0 & 0 & 0\\ 0 & 0 & 0.32 & 0 & 0\\ 0 & 0 & 0.35 & 0 & 0 \end{array}\right];$$

*b*_3 _= 0.64, *b*_5 _= 0.80, and all other *b*_*i *_= 0. Following the rules given by Remark 1, we are able to reconstruct the network shown in Figure 4. As the example shows, we are able to determine the interaction network given by (24) through the LP (20) even when degradation rates are considered uncertain.

### Reconstructing the glycolytic pathway of Lactococcus lactis

**Figure 5:**
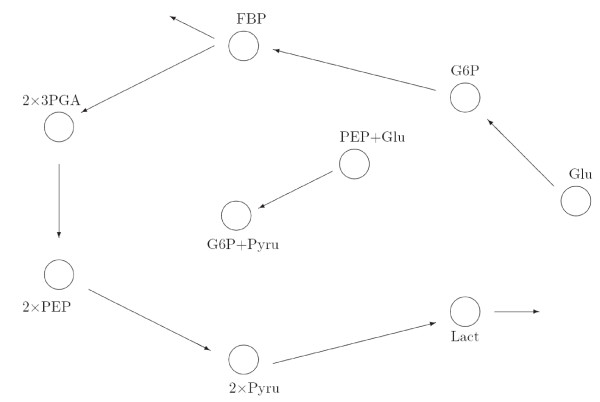
**The glycolytic pathway of *Lactococcus lactis***.

*Lactococcus lactis *is a bacterium used in the industrial production of cheese and buttermilk as it converts more than 90% of lactose (milk sugar) to lactic acid [14]. In general, the glycolytic pathway (or glycolysis) consists of chemical reactions that convert glucose into pyruvate. In the first step, glucose is converted into glucose-6-phosphate (G6P). A conversion of G6P into fructose-1,6-bisphosphate (FBP) follows, which is then converted sequentially to glyceraldehyde-3-phosphate (Ga3P), 3-phosphoglyceric acid (3-PGA) and PEP [16]. Additionally, Glucose and PEP are converted directly to pyruvate and G6P. Note that since measurement data for the intermediate Ga3P were unavailable, we include an additional rate denoting depletion of FBP. A simplified description of the pathway from reference [33] is depicted in Figure 5. The relative simplicity of this metabolic network makes *L. lactis *an attractive model for biological systems approaches [14]. A recent paper which presents an approach to determine the connectivity of this system and puts some emphasis on its sparsity is [16]. However, this approach does not take into account the characteristic particulars that make up a chemical reaction network. Here, we first use LP (11) to try to elucidate the glycolytic pathway of *L. lactis *using the same experimental data from [33].

Particularly, we wish to elucidate the glycolytic pathway of *L. lactis *under the assumption that the following complexes participate in the chemical reaction network: Glu, G6P, FBP, 2 × 3PGA, 2 × PEP, 2 × Pyru and Lact. In other words, we wish to obtain interaction topology *Aκ *of the chemical reaction network given by $\dot{x}$ = *Y A*_*κ *_*f *(*x*), where

$$Y=\left[\begin{array}{ccccccc} 1 & 0 & 0 & 0 & 0 & 0 & 0\\ 0 & 1 & 0 & 0 & 0 & 0 & 0\\ 0 & 0 & 1 & 0 & 0 & 0 & 0\\ 0 & 0 & 0 & 2 & 0 & 0 & 0\\ 0 & 0 & 0 & 0 & 2 & 0 & 0\\ 0 & 0 & 0 & 0 & 0 & 2 & 0\\ 0 & 0 & 0 & 0 & 0 & 0 & 1 \end{array}\right],x=\left[\begin{array}{c} \left[\text{Glu}\right]\\ \left[\text{G6P}\right]\\ \left[\text{FBP}\right]\\ \left[3\text{PGA}\right]\\ \left[\text{PEP}\right]\\ \left[\text{Pyru}\right]\\ \left[\text{Lact}\right] \end{array}\right],f(x)=\left[\begin{array}{c} \left[\text{Glu}\right]\\ \left[\text{G6P}\right]\\ \left[\text{FBP}\right]\\ {\left[3\text{PGA}\right]}^{2}\\ {\left[\text{PEP}\right]}^{2}\\ {\left[\text{Pyru}\right]}^{2}\\ \left[\text{Lact}\right] \end{array}\right].$$

Note that the network topology is completely determined by *A*_*κ*_. Recall that

(25) *A*_*κ *_= *K *- diag(*K*^T^*e*), *K*_*ij *_≥ 0 ∀*i*, *j*.

**Figure 6:**
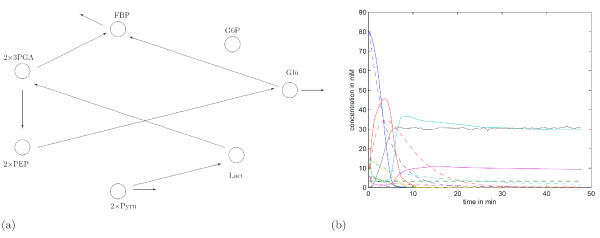
**Reaction pathway obtained through (11)**. a) The reaction pathway obtained through (11). b) The simulated model dynamics defined through the reaction network shown in (a) are shown in dashed lines and solid lines correspond to experimental data.

Now, by solving (11) we indeed obtain a sparse chemical reaction topology (Figure 6(a)). However, the error between the model dynamics and experimental data is unreasonably large (Figure 6(b)). Therefore, it is not surprising that this configuration differs greatly from the the proposed pathway of Figure 5.

**Figure 7:**
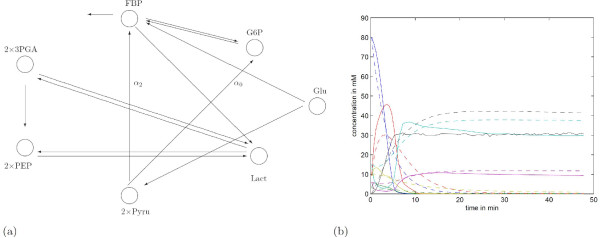
**Reaction pathway obtained through (26)**. a) Reaction pathway obtained through (26) for *α *= 0 and *α *= 2. All reactions participate in both pathways except for two which are marked accordingly. The one reaction that was obtained for *α *= 0 but not for *α *= 2 is marked with *α*_0 _and the one that appears only for *α *= 2 is marked with *α*_2_. b) Here, solid lines correspond to experimental data and dashed lines to the model with the interaction matrix obtained by solving (26) with *α *= 0.

The ℓ_1 _regularized least squares problem, which is called *Lasso *is statistics, considers an objective function to minimize, which consists of the sum of the 1-norm of the vector of unknowns and the least squares of the error:

(26) $$\begin{array}{ll} \text{given} & Y\\ \min & {\left\|\left[\begin{array}{c} \hat{x}(t_{1})-\hat{x}(t_{2})+(t_{2}-t_{1})Af(\hat{x}(t_{1}))\\ \vdots \\ \hat{x}(t_{p-1})-\hat{x}(t_{p})+(t_{p}-t_{p-1})Af(\hat{x}(t_{p-1})) \end{array}\right]\right\|}_{2}+\alpha ||\text{vec(}A\text{)}|{|}_{1}\\ \text{s}\text{. t}\text{.} & A=YA_{k},\\ & A_{\kappa_{i,j}}\geq 0,i\neq j,\forall i,j,e^{T}A_{\kappa }=0\;(\text{this follows from }(25)), \end{array}$$

where *α *is a nonnegative constant that allows us to regulate the weight we put on the sparsity of *A *explicitly. Note that for *α *= 0, program (26) minimizes the the error between data and model dynamics solely (Figure 7(b)). This time, the error between the model dynamics and experimental data is considerably smaller. The connection topology is shown in Figure 7(a). Now, we increase *α *to see whether or which interconnections disappear without altering the system dynamics significantly. This pathway, which remains unaltered for 2 ≤ *α *≤ 10, is shown in Figure 7(a). The dynamic behavior of this system is indistinguishable from the one shown in Figure 7(b) and, thus, is not shown.

Further increase of *α *results first in the disappearance of the links between G6P and FBP, and sequential changes do not result in 'sensible' connection topologies. Of course, this is something that in general the investigator does not know. While the pathway depicted in Figure 6(a) might be dismissed because the resulting model behavior compares badly with data, this argument does not hold for the pathway in Figure 7(a).

Now, we exploit the following related approach to try to deduce the interactions of the system by solving the following LP:

(27) $$\begin{array}{ll} \text{given} & Y\\ \min & {\left\|\left[\begin{array}{c} \hat{x}(t_{1})-\hat{x}(t_{2})+(t_{2}-t_{1})Af(\hat{x}(t_{1}))\\ \vdots \\ \hat{x}(t_{p-1})-\hat{x}(t_{p})+(t_{p}-t_{p-1})Af(\hat{x}(t_{p-1})) \end{array}\right]\right\|}_{1}+\alpha ||\text{vec(}A\text{)}|{|}_{1}\\ \text{s}\text{. t}\text{.} & A=YA_{k},\\ & A_{\kappa_{i,j}}\geq 0,i\neq j,\forall i,j,e^{T}A_{\kappa }=0\;(\text{follows from }(25)), \end{array}$$

**Figure 8:**
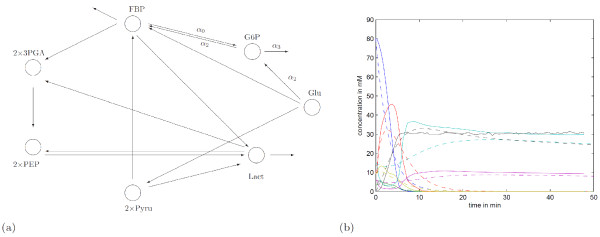
**Reaction pathway obtained through (27)**. a) All reactions participate in both pathways except for four which are marked accordingly. Two reactions that were obtained for *α *= 0 and *α *= 2 but not for *α *= 3 are marked with *α*_0 _and *α*_2_, one that appears only for *α *= 0 is marked with *α*_0_, and one that appears only for *α *= 3 is marked with *α*_3_. (Note that a gradual increase of *α*, for 3 ≤ *α *≤ 75, did not change the network structure.) b) Here, solid lines correspond to experimental data and dashed lines to the model dynamics defined through the reaction network shown in Figure 8(a) for *α *= 0.

We solve (27) for *α *= 0, *α *= 2 and *α *= 3, and obtain the reaction pathway shown in Figure 8(a) which results in a model with the dynamics depicted in Figure 8(b). (Note that for 0 <*α *≤ 75, the model dynamics are indistinguishable from the ones shown in Figure 8(b) and are not shown.)

The error between the model dynamics and experimental data is again considerably smaller than the error shown in Figure 6(b). As we can see from Figure 8(a), a relatively sparse reaction topology was obtained.

The pathway *x*_1 _→ ... → *x*_7 _was almost reconstructed. A sensible assumption to make is that the degradation of G6P which appears at *α *= 3 corresponds to the conversion into FBP suggested at *α *= 2.

Also, the direct link between glucose and pyruvate was discovered. Finally, with

$$A_{\kappa }=\left[\begin{array}{ccccccc} -0.3452 & 0 & 0 & 0 & 0 & 0 & 0\\ 0.0185 & -0.4105 & 0 & 0 & 0 & 0 & 0\\ 0.2431 & 0.4105 & -0.3735 & 0 & 0 & 0.0350 & 0\\ 0 & 0 & 0.0009 & -0.0008 & 0 & 0 & 0.0237\\ 0 & 0 & 0 & 0.0008 & -0.0106 & 0 & 0.0079\\ 0.0835 & 0 & 0 & 0 & 0 & -0.0377 & 0\\ 0 & 0 & 0.1551 & 0 & 0.0105 & 0.0027 & -0.0393 \end{array}\right]$$

our approach provides a meaningful chemical reaction network of the form (4). (This matrix corresponds to the case when *α *= 2.) Nevertheless, without biochemical information the superiority of this pathway to the pathway in Figure 7(a) cannot be established and it follows that experiments have to be designed to discriminate between several competing models.

## Conclusion

We have presented a methodology for determining the interaction topology of biological networks, that are either affine in the unknown parameters or can be transformed to have this property, using time series data collected from experiments. We demonstrated the ability of our method to identify a chemical reaction network structure through several numerical examples. We have also tested our approach by elucidating the glycolytic pathway of the bacterium *Lactococcus lactis*. Our method respects the structural properties that chemical reaction network dynamics should have [11,12].

In the case of gene regulatory networks, more realistic models could be used, but those would include additional parameters, first, by making the Hill coefficient in the activation and repression terms a free variable; and second, encoding the fact that when two transcription factors act on DNA, either both are required (AND) or any of them is sufficient (OR) for action. Thus, a valuable research direction is to investigate this case and establish whether similar analysis techniques to the ones presented in this paper can be used.

In (27) we introduced a free variable *α *whose value can change the solution considerably. Hence, it is worthwhile to explore different possible heuristics how to choose the value of this variable. (Here, we kept the balance between increasing *α *and keeping the model dynamics that followed from the solution of (27) relatively close to experimental data.) An iterative method can also be used, which uses 'live' information from simulations and then iterates with a simple Linear Program to find the network structure that fits best the parameters.

Finally, as shown, different methods or the same one with different constraints provide different models that represent the same data, which is an expected feature of such methods. It follows that experiments have to be designed to discriminate between competing models, in a way that 'closes the loop' between modelling and experiment (see for example [31]).

## Authors' contributions

The authors contributed equally to this work. The first author developed the algorithm for determining gene regulatory networks and performed the example of *L. Lactis *while the second author developed the algorithm for biochemical reaction networks and conceived the general idea. Both authors read and approved the final manuscript.
